# Frequency and predictors of pulmonary hypertension in patients with Systemic Lupus Erythematosus

**DOI:** 10.12669/pjms.35.1.405

**Published:** 2019

**Authors:** Sadia Asif, Aflak Rasheed, Tafazzul-e-Haque Mahmud, Ammad Asghar

**Affiliations:** 1 Dr. Sadia Asif, FCPS (Medicine), Fellow Rheumatology, Department of Rheumatology, Sheikh Zayed Hospital, Lahore, Pakistan; 2 Dr. Aflak Rasheed, FCPS (Medicine), FCPS Rheumatology, Department of Rheumatology, Sheikh Zayed Hospital, Lahore, Pakistan; 3 Dr. Tafazzul-e-Haq Mahmud, MB MRCP (UK) FRCP (London), Department of Rheumatology, Sheikh Zayed Hospital, Lahore, Pakistan; 4 Dr. Ammad Asghar, FCPS (Medicine), PGR Rheumatology, Department of Rheumatology, Sheikh Zayed Hospital, Lahore, Pakistan

**Keywords:** Mean Pulmonary Arterial Pressure (MPAP), Pulmonary Arterial Hypertension (PAH), Pulmonary Artery Systolic Pressure (PASP), Systemic Lupus Erythematosus (SLE)

## Abstract

**Objective:**

To determine the frequency and predictors of pulmonary hypertension in patients with Systemic Lupus Erythematosus in a Pakistani population, presenting at a tertiary care hospital

**Methods:**

This cross-sectional study was conducted at the Department of Rheumatology, Shiekh Zayed Hospital, Lahore from March to June 2018. A total of 97 patients, who fulfilled the Systemic Lupus Erythematosus (SLE) criteria of American College of Rheumatology (ACR) 1992 were enrolled. Pulmonary Arterial Hypertension (PAH) was measured by calculating pulmonary arterial systolic pressure through echocardiography by a single consultant cardiologist. Disease characteristics and demography was collected in a self-administered proforma. PAH was defined as mean pulmonary arterial pressure of 25mmHg or above by calculating with a formula. SPSS version 20 was used for analysis of data.

**Results:**

Out of 97 patients, 89.7% (n=87) were females and 10.3% (n=10) were males, with mean age of 31.29± 8.824 years. The mean disease duration was 24.21 ± 30.46 months. PAH was found in 23.3% (n=23) patients, including 19 females and 4 males. On further analysis of data, Raynaud phenomenon, rheumatoid factor and nephritis were assessed as predictors of PAH and all of these showed statistical significance for presence of PAH as per Chi-square test (p<0.05).

**Conclusion:**

In this study, 23.3% SLE patients showed evidence of PAH and positive statistical significance was found between predictors like Raynaud phenomenon, rheumatoid factor, nephritis and presence of PAH. So it is imperative to detect PAH early and start prompt treatment to achieve better quality of life.

## INTRODUCTION

Systemic Lupus Erythematosis (SLE) is an autoimmune disorder and a multisystem disease.^1^ On one end it can cause rash, arthritis and fatigue and on the other end it can cause nephritis, thrombocytopenia, anemia, serositis and neurological problems.^1^ SLE is diagnosed in patients who fulfil 4 out of the 11 diagnostic ACR 1992 revised and updated criteria.^2^ One of the manifestations in SLE is Pulmonary arterial Hypertension (PAH) even though it’s not a diagnostic criteria, it comes under one of the fatal manifestations.^3^ Pulmonary arterial Hypertension (PAH) associated with connective tissue disease has a poor prognosis, although patients with SLE with PAH have better prognosis as compared with those with scleroderma with PAH.^3^ Even though PAH has disastrous consequences in rheumatic diseases, its frequency is not well studied in Lupus.^4^ In one study, the frequency of PAH in SLE ranged between 0.5 to 17.5%.^5^

There are several predictors for development of pulmonary hypertension in SLE patients such as serositis (pleural effusion and pericardial effusion), raynaud’s phenomenon, nephritis and rheumatoid factor.^5^ Although it has been noticed that SLE patients with serositis, nephritis, rheumatoid factor and anti-cardiolipin antibodies were found to have pulmonary hypertension rather than those who did not have such complications.^5^ Conclusively, it can be suggested that early treatment initiation in SLE patients with above mentioned predictors can lead to favorable outcome regarding prognosis.^5^ Pulmonary hypertension in SLE is multifactorial, genetics, environment and immune system contribute to play a significant role.^6^ All these factors create an imbalance between vasoconstrictors and vasodilator substances leading to increased pulmonary vascular resistance (PVR).^6^ The levels of thromboxane and endothelins are significantly raised in patients of SLE with pulmonary hypertension as compared to those SLE patients who don’t have pulmonary hypertension.^6^ About 42% of patients with SLE with pulmonary arterial hypertension were found to have antibodies against the endothelin receptor Type-A. Also small microthrombi formation occurs into the vessels, subsequently precipitating further increase in PASP.

There are several confounding factors which can cause PAH, such as interstitial lung disease, ischemic heart disease, valvular heart disease, cardiomyopathy and COPD, or PAH caused by other connective tissue disease. So, these were excluded from the study.^7^ In our population, due to the delayed presentation of disease, complicated cases are seen in clinics.

Aim of this study was to determine the frequency of pulmonary hypertension in SLE patients of Pakistani origin using echocardiography which is non-invasive method and do not require specialized training and can be used in routine clinical practice.^8^ We want to make a comparison of frequency of pulmonary arterial hypertension in SLE patients in Pakistan with rest of the world as severity of disease varies in different populations. No such study has been done previously in Pakistan so we want to conduct this study to draw attention of physicians to find such unnoticed complications of SLE.

## METHODS

This cross-sectional, observational study was conducted in Department of Rheumatology, Shiekh Zayed Hospital, Lahore, from March to June 2018, after approval from Institutional Review Board (IRB), Shiekh Zayed Hospital, Lahore.

A total of 97 patients were selected from both outpatient and inpatient department after sample size calculation (95 % Confidence level, 05 % margin of error and taking frequency of pulmonary arterial hypertension in Systemic Lupus Erythematosus of 23%. Patients who fulfil 4 out of the 11 diagnostic ACR 1992 revised and updated criteria for Systemic Lupus Erythematosus were included in this study. Excluded were those patients who had ischemic heart disease, valvular heart disease or cardiomyopathy. Patients with mixed connective tissue disease or having history of smoking or COPD or ILD were also excluded. Each patient was required to sign written informed consent and confidentiality was maintained. Demographic data and disease variables were recorded in a proforma.

Echocardiography was performed for calculation of pulmonary arterial hypertension by single consultant cardiologist, Sheikh Zayed Hospital, Lahore. Rest of the investigations (chest x-ray, complete urine examination, 24 hour urinary proteins and renal function tests, rheumatoid factor) pertinent to determination of the predictors of SLE/PAH were advised. Echocardiography with Doppler studies of the tricuspid and pulmonary valves was performed to confirm diagnosis of pulmonary hypertension. Mean pulmonary artery pressure (MPAP) was calculated by the following formula MPAP = 0.61 x PASP + 2 mm Hg and patient was labelled as having PAH if MPAP ≥25mmHg.

All this information was analysed using SPSS version 20. The presence of PAH and predictors associated with PAH were calculated as frequencies and percentages. Two groups were made, one with patients having PAH and without PAH. For both stratified groups frequency of PAH and predictors of pulmonary arterial hypertension was calculated and significance was checked by using Chi-square (*x^2^*) test.

## RESULTS

**Fig.1:**
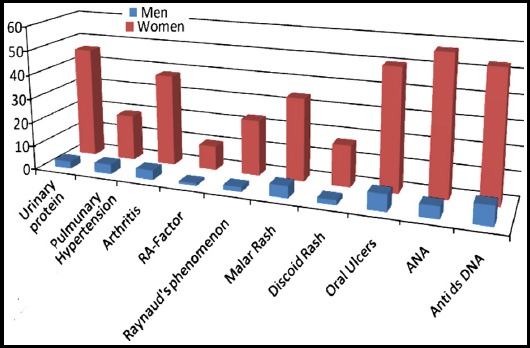
Frequency of various clinical and biochemical features in patients with SLE

In this study, 98 patients of SLE were taken. Out of 98 enrolled SLE patients, 88 were females and 10 were males with a mean of 31.29 ± 8.824. The frequency of pulmonary hypertension among them was 23.3%. According to SLE criteria, various features i.e. proteinuria, serositis, oral ulcers and arthritis etc. were also studied. Their percentages and relative gender distribution has been shgown in Fig.1.

Strong correlation of PAH in SLE patients with various predictors are presented in

Table-I suggesting a future plan to work on it in detail.

**Table-I:** Association of various factors with pulmonary hypertension in SLE

VARIABLES	Pulmonary Hypertension		P-Value

	Yes	No	
***Raynaud’s Phenomenon***			
Present	11	61	0.005
Not Present	12	14	
***Urinary Proteins***			
Detected	19	30	<0.001
Not Detected	4	45	
***RA-Factor***			
Positive	7	4	0.001
Negative	16	71	

The predictors found to have association with pulmonary arterial hypertension in SLE patients were raynaud’s phenomenon, urinary proteins and rheumatoid factor, with a significant p-value (<0.05) Table-II.

**Table-II:** Frequency distribution of all variables.

Variables	Detected/Present/Positive			Not Detected/Absent/Negative		

	Total	Male	Female	Total	Male	Female
Urinary Protein	49(50%)	3	46	49(50%)	7	42
Pulmonary Hypertension	23(23.3%)	4	19	75(76.7%)	6	69
Arthritis	42(42.9%)	4	38	56(57.1%)	6	50
RA- Factor	11(11.2%)	1	10	87(88.2%)	9	78
Raynaud’s Phenomenon	25(25.5%)	2	23	73(74.5%)	8	65
Malar Rash	39(39.8%)	5	34	59(60.2%)	4	54
Discoid Rash	19(19.4%)	2	17	79(80.6%)	8	71
Oral Ulcers	57(58.1%)	7	50	41(41.9%)	3	38
ANA	62(63.2%)	5	57	36(36.8%)	5	31
Anti ds DNA	61(62.2%)	8	53	37(37.8%)	2	35

## DISCUSSION

Systemic lupus erythematosus (SLE) is an autoimmune chronic inflammatory disease that has protean manifestations involving multiple organs of body. Pulmonary and cardiac manifestations are common in SLE patients, which are common cause of mortality and morbidity.^8^ Pulmonary arterial Hypertension is the least studied parameter among patients o SLE, but it is not uncommon.^8^ Diagnosis of early PAH can safeguard patients from grave consequences.^8^

According to this study, a total of 97 patients were taken and the frequency of pulmonary hypertension was found to be 23.3% in that group.^9^ To the best of my knowledge there is no local study published on this topic.

According to another study, frequency of pulmonary hypertension was found to be 1-14%.^10^ In one study, frequency of PAH in SLE was found to be 10.8% and in those patients rheumatoid factor and anti-cardiolipins were found high.^8^ In another study prevalence of PAH in SLE patients ranges from 0.5% to 17.5%.^5^ In another Chinese study of 642 patients, prevalence of pulmonary hypertension was found to be between 5 -14%.^9^ These results are almost comparable to our data.

Among all connective tissue diseases, SLE is the second most common disease after systemic sclerosis in with PAH is common and affect disease prognosis.^11^ The prevalence of PH in SLE was 2.8–23.3%.^9^ A comparative study was conducted in which various pulmonary manifestations in SLE patients have been studied and pulmonary hypertension was found to be one of the important complication in such patients.^12^ In another meta-analysis of various studies, it was found that PAH was found in 8% of SLE patients.^13^ Various predictors of PAH are compared which are found directly with PAH, such as nephritis, serositis (pleural and pericardial effusion), raynaud phenomenon and rheumatoid factor). Raynaud phenomenon is an important risk factor to determine PAH in SLE patients.^14^ Patients without pleuritis and pericarditis are at low risk of PAH.^15^ Several studies indicated that serositis, nephritis, raynaud phenomenon and rheumatoid factor are independently related with development of PAH in SLE patients.^16^ The present study proved the relationship of raynaud’s, nephritis and rheumatoid factor with PAH in SLE patients.

On the basis of publications reporting correlations with invasive measurement data, Doppler echocardiography is still recommended as the primary tool for early screening and assessment of patients with clinical suspicion of PAH.^17^ According to the latest guidelines, a detailed echocardiographic assessment is equally useful diagnostic modality for the detection of PAH.^18^ And it is non-invasive method and easy to perform in SLE patients.^19^

## CONCLUSION

We conclude that frequency of PAH in SLE patients is significantly high and in the presence of observed predictors we should strongly suspect and recommend for early screening for PAH. This will help in early diagnosis, better treatment and reducing the complications.

### Author’s Contribution

**SA, AR & THM** conceived the manuscript.

**SA** designed the manuscript, did data collection, statistical analysis and manuscript writing & editing.

**AA** did review and final approval of manuscript.
